# 조기폐경 여부에 따른 삶의 질 관련 요인: 국민건강영양조사 자료(2014-2017년) 분석

**DOI:** 10.4069/kjwhn.2020.03.27

**Published:** 2020-04-10

**Authors:** Ok-Hee Cho, Kyung-Hye Hwang

**Affiliations:** 1 Department of Nursing, College of Nursing and Health, Kongju National University, Gongju, Korea; 1 공주대학교 간호학과; 2 Department of Nursing, Suwon Science College, Hwaseong, Korea; 2 수원과학대학교 간호학과

**Keywords:** Health behavior, Menopause, Menopause premature, Quality of life, 건강행동, 폐경, 조기폐경, 삶의 질

## Abstract

**Purpose:**

The purpose of this study was to compare the health‐related quality of life of premature menopausal women with that of normal postmenopausal women and to identify factors related to quality of life in these two groups.

**Methods:**

A secondary analysis was conducted of a sample of 5,910 postmenopausal women drawn from women aged 19–79 years, using raw data from the Korea National Health and Nutrition Survey between 2014 and 2017. Quality of life was compared between premature menopausal women and normal postmenopausal women using the t‐test and chi‐square test, and factors related to quality of life were analyzed by multivariate logistic regression using SAS version 9.4.

**Results:**

There was no significant difference in quality of life between premature menopausal women and normal postmenopausal women. Subjective health status and symptoms of depression were identified as factors related to quality of life in women with premature menopause, while engaging in aerobic physical activity, subjective health status, stress, and symptoms of depression were found to be related to quality of life among normal postmenopausal women.

**Conclusion:**

It is necessary to support self‐care measures for both premature and normal menopausal women to adapt to changes caused by menopause, and multidimensional strategies should be developed considering postmenopausal women’s subjective health status and symptoms of depression to improve their quality of life.

## Introduction

### 연구 필요성

조기폐경은 40세 이전에 폐경이 되는 것으로 [1] 무월경 여성 중 10–28%에서 나타나는 것으로 보고되고 있다[2]. 우리나라에서 40세 이전에 폐경 장애로 진료를 받은 여성은 2014년 3,575명에서 2018년 약 6,300명으로 증가하는 추세에 있다[3].

조기폐경은 자연적으로 발생하는 일차성 폐경과 항암치료, 방사선요법, 수술적 난소절제술 등으로 인한 이차성 폐경으로 구분될 수 있으며, 유전, 조기 난소 부전, 자가면역, 대사, 감염, 원인불명의 난소증후군, 시상하부 장애 등으로 발생할 수 있다[1]. 조기폐경 여성들은 월경의 중단과 함께 에스트로겐의 감소로 비뇨생식기의 위축과 성기능 장애가 나타나며[4], 탈모, 안구 건조, 사지 저림, 저혈압[5], 심혈관 질환, 관절통, 골밀도 저하 등의 신체 증상을 경험하고[6,7], 가임 능력의 상실로 여성성이 박탈됨을 느끼면서 절망감과 주변의 시선을 의식하여 위축된다[8]. 조기폐경 여성은 폐경 후에 인지 및 정서 장애가 발생할 수 있으나, 선행연구에서는 자연적으로 조기폐경이 된 경우가 수술과 항암치료 등으로 인해 인공폐경이 된 경우보다 우울 정도가 높았다고 보고하였다[9].

한편 폐경 후에는 체질량지수가 증가하여 복부 비만, 고혈압이 발생하고[10] 갑상선 저하증, 당뇨 등의 만성질환 발생 위험 또한 높다[1]. 따라서 폐경 후의 건강 행태는 삶의 질을 개선하는 데 주요 원인이 된다[11]. 폐경의 연령이 낮을수록 스트레스 정도가 높았던 반면에[10] 신체적으로 활동적인 여성이 신체 증상 정도를 더 적게 경험하였으며[12], 흡연 여성이 비흡연 여성보다 조기폐경 발생이 많았다고 보고되었다[13]. 조기폐경 후 나타나는 신체적, 정신적 증상들과 건강 행태는 삶의 질을 위협하는 부정적인 요인으로 작용할 수 있다[14,15].

조기폐경 여성은 주변의 시선을 의식하여 심리적으로 위축되고[8] 질병을 관리할 자신감이 많이 떨어짐에 따라[9] 폐경기 증상 관리에 신경을 덜 쓰는 경향이 있다[8]. 조기폐경 여성은 신체적 건강과 심리적 영역에서 삶의 질이 저하될 위험이 2.85배 더 높은 것으로 나타났으며[14], 신체적 건강[12], 건강 행태[14], 우울[9], 흡연[13] 등의 요인이 관련됨을 보고하였다.

이제까지의 조기폐경 여성에 대한 국내 선행연구들은 조기폐경 발생 요인에 대한 연구가 대부분으로[6,16], 조기폐경 여성과 정상폐경 여성의 삶의 질의 비교와 관련 요인을 파악한 연구는 부족한 실정이다. 이에 본 연구는 전국 표본 집단을 대상으로 실시한 국민건강영양조사 자료(2014–2017년)를 활용하여 조기폐경 여부에 따른 삶의 질의 차이를 비교하고, 조기폐경 여성과 정상폐경 여성의 삶의 질 관련 요인을 살펴보고자 하였다.

### 연구 목적

본 연구의 목적은 우리나라 여성의 조기폐경 여부에 따른 삶의 질 비교 및 관련 요인을 파악하기 위함이며, 구체적인 목적은 다음과 같다.

1) 조기폐경 여부에 따른 일반적 특성을 비교한다.

2) 조기폐경 여부에 따른 건강행태와 건강상태를 비교한다.

3) 조기폐경 여부에 따른 삶의 질 차이를 비교한다.

4) 조기폐경 여부에 따른 삶의 질 관련 요인을 파악한다.

## Methods

Obtaining informed consent was exempted by the Institutional Review Board (IRB) of Kongju National University (IRB-2019-11) because the data were from the Korea National Health and Nutrition Survey (https://knhanes.cdc.go.kr/).

### 연구 설계

본 연구는 국민건강영양조사 원시자료를 활용하여 조기폐경 여부에 따른 삶의 질 비교 및 관련 요인을 파악하기 위한 상관성 조사연구 설계이다.

### 연구 대상

본 연구에서는 제6기 2차년도(2014–2015년)부터 제7기 2차년도(2016–2017년)까지의 국민건강영양조사 원시자료를 이용하여 이차 분석하였다. 총 응답자는 36,545명이었으며, 이 중 여성은 20,148명이었다. 조기폐경 여성과 정상폐경 여성의 비교를 위해 19–79세의 여성을 선별하였고, 응답자 15,322명 중 ‘현재 월경(생리, 달거리)을 하고 있습니까?’라는 질문에 ‘자연폐경’ 또는 ‘인공폐경’이라고 응답한 5,910명의 여성을 대상으로 하였다.

### 연구 도구

#### 폐경

본 연구에서 폐경 여부는 40세 이전에 폐경이 된 경우를 조기폐경, 40–58세에 폐경이 된 경우를 정상폐경으로 분류하였다[17,18].

#### 신체건강, 정신건강 및 건강 행태

신체건강은 주관적 건강 상태와 의사에게 진단받은 고혈압과 당뇨 유무를 파악하였고, 주관적 건강 상태는 ‘좋음’(매우 좋음 또는 좋음), ‘보통’, ‘나쁨’(나쁨 또는 매우 나쁨)으로 분류하였다.

정신건강은 스트레스 정도와 우울 증상을 파악하였다. 스트레스 정도는 평소 일상생활 중에 스트레스를 ‘적게 느끼는 경우’와 ‘많이 느끼는 경우’로 분류하였고, 우울 증상은 최근 1년 동안 2주 이상 연속 ‘우울감이 없는 경우’와 ‘우울감이 있는 경우’로 분류하였다.

건강 행태는 흡연과 폭음 여부, 유산소 신체활동 실천율로 파악하였다. 흡연은 현재 흡연의 경우로, 폭음은 한 달에 1번 이상 한 번의 술자리에서 술잔 5잔 이상 마시는 경우로, 유산소 신체활동은 일주일에 중강도 신체활동을 2시간 30분 이상, 또는 고강도 신체활동을 1시간 15분 이상, 또는 중강도와 고강도 신체활동을 섞어서 각 활동에 상당하는 시간을 실천하는 경우로 정의하였다.

#### 삶의 질

삶의 질은 EuroQoL Group이 개발한 Euro­Qol-5 Dimension (EQ-5D)로 측정하였다. EQ-5D는 운동 능력, 자기 관리, 일상 활동, 통증/불편감, 불안/우울 등 5개 항목으로, 각 항목에 대해 ‘지장이 없다’, ‘다소 지장이 있다’, ‘심하게 지장이 있다’로 응답하도록 되어 있다. 삶의 질 지수(EQ-5D index score)는 질병관리본부에서 실시한 질 가중치 연구[19]에서 도출된 보정식을 이용하여 완전한 건강 상태 1부터 가장 낮은 점수 –0.171까지의 가중지표 값으로 계산하였고, 점수가 높을수록 삶의 질이 좋음을 의미한다. ‘삶의 질 저하’를 정의할 수 있는 절단점이 정해져 있지 않아 선행연구[20]에 근거하여 EQ-5D index score의 가장 낮은 5분위수를 삶의 질 저하의 절단점으로 임의로 정하여 관련 요인을 파악하였다.

#### 일반적 특성

연령, 결혼 여부, 직업, 소득, 교육 정도, 거주 지역 등 사회경제적 특성 6문항과, 폐경 원인, 폐경 후 기간 등 폐경 관련 특성 2문항을 파악하였다. 직업은 ‘사무직’, ‘현장직’, ‘무직(전업 주부 포함)’으로, 소득은 가구 소득금액의 사분위수를 기준으로 ‘하’, ‘중하’, ‘중상’, ‘상’으로, 교육 정도는 ‘초등학교 졸업 이하’, ‘중학교 졸업’, ‘고등학교 졸업’, ‘대학교 졸업’으로 분류하였고, 거주 지역은 특별시와 광역시는 ‘도시’로, 나머지 지역은 ‘비도시’로 분류하였다.

### 자료 분석 방법

수집된 자료는 SAS 프로그램 version 9.4 (SAS Institute, Cary, NC, USA)을 이용하였고, 복합표본설계를 위해 질병관리본부의 국민건강영양조사 원시자료 이용지침에 따라 가중치를 사용하여 분석하였다. 조기폐경군과 정상폐경군의 인구사회학적 특성, 건강 행태 특성, 신체건강과 정신건강 특성, 삶의 질의 차이는 실수와 백분율, 평균, 표준편차로 파악하였다. 조기폐경군과 정상폐경군의 인구사회학적 특성, 건강 행태 특성, 신체건강과 정신건강 특성, 삶의 질의 차이는 t-test와 Rao-Scott chi-square test로 분석하였으며 실수와 백분율, 평균, 표준편차로 파악하였다. 조기폐경군과 정상폐경군 각각의 삶의 질 관련 요인을 알아보기 위하여 인구사회학적 특성을 공변량으로, 건강 행태 특성, 신체건강과 정신건강 특성을 독립변수로, ‘낮은 삶의 질 지수(lowest 5th quintile of EQ-5D index score)’를 종속변수로 투입하여 다변량 로지스틱 회귀분석을 시행하였다.

## Results

### 조기폐경 여부에 따른 일반적 특성의 차이

대상자 중 40세 이전에 조기폐경이 된 여성은 총 322명(5.4%)이었고, 40–58세에서 폐경이 된 정상폐경 여성은 총 5,588명(94.6%)이었다. 조사 시점 기준에서 조기폐경군 중 50세 미만은 20.7%였으며, 정상폐경군에서는 4.4%였다(*p*<.001). 조기폐경군에서 초등학교 졸업자는 46.1%였고 정상폐경군에서는 41.9%였다(*p*=.018). 조기폐경군의 65.3%가 인공폐경이 원인이었고, 정상폐경군의 87.9%가 자연폐경이 원인이었다(*p*<.001). 조기폐경군에서 폐경 후 10년 이상 지난 경우는 91.4%였고, 정상폐경군에서는 53.7%였다(*p*<.001) (Table 1).

### 조기폐경 여부에 따른 건강 행태, 신체건강 및 정신건강 특성의 차이

조기폐경 여부에 따른 대상자의 건강 행태 특성의 차이는 흡연을 하는 경우가 조기폐경된 군에서 10.2%, 정상폐경군에서 3.6%였고(*p*<.001), 폭음, 유산소 신체활동 실천율은 통계적으로 유의한 차이가 없었다.

대상자의 신체건강 특성의 차이는 주관적 건강 상태가 ‘나쁨’과 ‘매우 나쁨’이 조기폐경군에서 36.3%, 정상폐경군에서는 27.2%였고(*p*=.011), 당뇨가 있음이 조기폐경군의 17.8%, 정상폐경군에서는 13.0%였다(*p*=.025). 대상자의 정신건강 특성의 차이는 우울 증상이 있는 경우가 조기폐경군에서 12.8%, 정상폐경군에서는 8.3%였고(*p*=.010), 스트레스에 따른 통계적으로 유의한 차이는 없었다(Table 2).

### 조기폐경 여부에 따른 삶의 질의 차이

조기폐경군의 삶의 질은 0.89점, 정상폐경군은 0.91점으로 통계적으로 유의한 차이가 없었다. 하부 영역인 운동 능력, 자기 관리, 일상생활, 통증/불편, 불안/우울에서도 두 군 간에 통계적으로 유의한 차이가 없었다(Table 3).

### 조기폐경 여부에 따른 삶의 질 관련 요인

조기폐경 여부에 따라 차이가 있었던 결혼 상태, 직업, 수입, 교육, 지역, 폐경 원인, 폐경 후 기간 등 일반적 특성을 보정하여 조기폐경군과 정상폐경군의 ‘삶의 질 저하’ 발생 관련 요인에 대해 각각 다변량 로지스틱 회귀분석을 시행하였다. 조기폐경군 삶의 질의 로지스틱 회귀 모형은 유의하였으며(likelihood ratio χ2=114.07, *p*<.001), Nagelkerke R^2^=.31, max-rescaled R^2^=.47이었다. 영향 요인으로는 주관적 건강 상태(*p*=.013), 우울 증상(*p*=.002) 등 2개 요인이 확인되었다. 정상폐경군의 삶의 질 로지스틱으로 변경 회귀 모형은 유의하였으며(likelihood ratio χ2=1,268.90, *p*<.001), Nagelkerke R^2^=.22, max-rescaled R^2^=.34였다. 영향 요인으로는 유산소 신체활동 실천(*p*=.001), 주관적 건강 상태(*p*<.001), 스트레스(*p*<.001), 우울 증상(*p*<.001) 등 4개 요인이 확인되었다(Table 4). 즉, 조기폐경군에서는 주관적 건강 상태가 ‘나쁨’인 경우가 ‘좋음’인 경우에 비해 삶의 질이 저하될 위험이 5.49배 높았으며, 우울 증상이 있는 경우가 없는 경우에 비해 삶의 질이 저하될 위험이 4.25배 높았다. 정상폐경군에서는 유산소 신체활동을 실천하지 않는 경우가 실천하는 경우에 비해 삶의 질이 저하될 위험이 1.35배 높았고, 주관적 건강 상태가 ‘보통’인 경우와 ‘나쁨’인 경우가 ‘좋음’인 경우에 비해 각각 2.28배, 9.42배 높았다. 스트레스가 있는 경우와 우울 증상이 있는 경우가 그렇지 않은 경우에 비해 삶의 질이 저하될 위험이 각각 1.62배, 1.67배 높았다(Table 4).

## Discussion

본 연구는 2014년에서 2017년까지 4년간의 국민건강영양조사 자료를 활용하여 조기폐경 여성과 정상폐경 여성의 삶의 질 관련 요인을 파악하였다. 본 연구에서 40세 이전에 조기폐경군은 5.4%였는데, 이는 2007–2008년의 국민건강영양조사 자료를 분석한 연구에서 2007년 40세 이전의 조기폐경 여성이 9.4%인 결과[16]보다는 낮았다. 조기폐경군(46.1%)에서 정상폐경군(41.9%)보다 초등학교 졸업자 비율이 다소 높았는데, 이는 Choi 등[17]의 연구와 Ortega-Ceballos 등[21]의 연구에서 조기폐경 여성의 교육수준이 정상폐경 여성보다 낮았다고 한 결과와 유사하였다. 교육수준과 조기폐경의 관련성을 설명하기는 무리가 있으나, 교육수준이 낮은 폐경 여성은 폐경기에 대해 더 스트레스가 많으며[17], 폐경 여성의 낮은 교육수준은 활동과 일상생활에 영향을 미쳐 삶의 질에 부정적인 영향을 줄 수 있기 때문에[22], 조기폐경 여성에게 폐경 후 일상생활과 신체활동 교육을 제공할 때는 눈높이 교육을 제공하면 이해 부족에서 오는 걱정과 불이행 정도를 감소시킬 수 있다고 생각된다. 또한 Cha [16]의 연구에서 조기폐경군 중 일차성 조기폐경군이 86.7% (287명/331명)인데 반해, 본 연구는 조기폐경군 중 자연폐경이 34.7%로 추후 조기폐경의 원인에 대해 반복 연구할 필요가 있다.

본 연구에서 조기폐경군의 65.3%가 인공폐경이 원인이었고, 정상폐경군의 87.9%가 자연폐경이 원인이었다. 이는 Franić-Ivanišević 등[2]의 연구에서 조기폐경 여성 중 10–28%가 일차성 폐경(자연폐경), 4–18%가 이차성 폐경(인공폐경)으로 보고한 결과[2]보다 더 높은 비율이다. 질환의 치료로 유발되는 인공적인 조기폐경은 심혈관 질환, 신경 장애, 심리적 장애와 골다공증의 위험을 증가시키는 요인이 될 수 있다[23]. Allshouse 등[5]의 연구에서는 조기폐경 여성의 26%가 조기폐경 진단전에 우울 증상을 5년 이상 경험하였으며, 정상폐경에 비해 조기폐경 여성은 정서 변화, 탈모, 단순 관절음(joint clicking) 등의 증상이 시간이 지나도 줄어들지 않음을 보고하였다. 또한 조기폐경군에서 자연폐경인 경우가 34.7%나 되는 점을 고려할 때, 자연폐경으로 조기폐경된 여성에 대한 신체적, 정서적 사정과 관리가 필요하다고 하겠다.

본 연구의 조기폐경군에서 폐경 후 10년 이상 지난 경우는 91.4%였고 정상폐경군에서는 53.7%였다. Allshouse 등[5]의 연구에서 조기폐경 여성은 정상폐경 여성에 비해 폐경 후 긴 시간이 지나도 폐경 증상이 줄어들지 않는다고 보고하여, 정기적 관리의 필요성을 추정할 수 있다. 조기폐경은 갱년기 증후군을 경험하게 하고, 장기적인 관점에서는 성적 기능의 감소와 더불어 퇴행성 질환, 심혈관 질환 등으로 신체적 건강과 심리적 고통의 위험이 증가하게 되며[4], 조기폐경 후 폐경 기간이 길어지면 건강 문제 발생 위험이 증가할 수 있기 때문에[23], 조기폐경 진단 후에 정기적으로 진료 및 검사를 받는 것이 중요하다.

본 연구에서 흡연은 조기폐경군에서 10.2%, 정상폐경군에서 3.6%로 차이가 있었다. 선행연구에서도 정상폐경군에 비해 조기폐경군에서 현재와 과거 흡연의 비율이 높았고[16], 흡연을 하는 경우 정상폐경군에 비해 조기폐경군의 스트레스가 높았으며[17], 흡연은 조기폐경과 밀접하게 관련됨을 보고하였다[13]. 이런 근거로 조기폐경군 여성에게 간호를 제공할 때는 흡연력 및 현재 흡연 여부를 확인하고 금연 교육과 스트레스 관리를 동시에 진행하는 개별적인 접근이 요구된다.

본 연구에서 주관적 건강 상태가 ‘나쁨’과 ‘매우 나쁨’이 조기폐경군에서 36.3%, 정상폐경군에서는 27.2%였다. 이는 Cha [16]의 연구에서 조기폐경군의 주관적 건강 상태가 ‘나쁨’과 ‘매우 나쁨’이 조기폐경군에서 39.6%, 정상폐경군에서 12.4%로 나타난 결과와 유사하였다. 인지된 나쁜 주관적 건강 상태는 폐경 증상 뿐 아니라 사회적 환경[24], 정서 상태와 대인관계를 낮추는 요인으로 질병의 발생과 밀접한 관련이 있어[25], 주관적 건강 상태 인식을 건강 위험의 신호로서 관심을 가지고 관리할 필요가 있다. 또한 본 연구에서 당뇨가 조기폐경군의 17.8%, 정상폐경군에서는 13.0%였는데, 네덜란드의 조기폐경 여성이 정상폐경 여성보다 제2형 당뇨병의 발생위험이 4배 높다는 보고가[26] 본 연구의 결과를 뒷받침한다. 당뇨가 폐경 전에 이미 존재하고 있었는지 알 수 없지만 조기폐경 후 추적 검진과 건강 증진을 위한 일상생활 관리가 더욱 중요하다고 하겠다. 그러나 조기폐경 여성은 질병 관리에 대한 신념이 감소하는 경향이 있으므로[9], 간호 사정시 갱년기 증상과 동반되는 만성질환 유무와 관리 방법에 대해서도 적극적인 관심을 가져야 하겠다.

본 연구에서 우울 증상이 있는 경우가 조기폐경군에서 12.8%, 정상폐경군에서는 8.3%였고, 스트레스에 따른 차이는 없었다. 이는 조기폐경 여성의 우울 정도가 폐경 전 여성보다 높았고[9], Choi 등[17]의 연구에서는 조기폐경 여부에 따른 우울 증상의 차이는 없었지만 조기폐경군이 정상폐경군에 비해 스트레스를 많이 느끼는 것으로 나타나 본 연구 결과와 달랐다. 조기폐경 여성은 이른 나이에 조기폐경이라는 사실을 감당하기 어려워 모든 일에 의욕을 가지지 못하고 우울한 감정을 호소하며, 감정 조절이 안되어 자살 충동을 느끼는 경우도 있었다[8]. 특히 난소 부전으로 인해 조기폐경된 여성의 우울이 폐경 전 여성에 비해 높았다[9]. 이는 여성성을 조기에 상실한 것에 대한 좌절감이 우울한 기분을 높인 것으로 생각되며, 조기폐경 여성의 정서 변화에 우선적으로 관심을 가지고 일상생활 활동을 조절하도록 지원할 필요가 있다.

본 연구에서 조기폐경군과 정상폐경군 간에 삶의 질의 차이는 없었다. 이는 선행연구에서 조기폐경군이 정상여성군에 비해 낮은 삶의 질을 경험한 결과[15]와는 달랐다. Benetti-Pinto 등[14]의 연구에서 조기폐경 여성과 정상 여성 사이에 전체적인 삶의 질의 차이는 없는 것으로 나타났다. 이렇게 다른 결과는 본 연구에서 사용한 EQ-5D 삶의 질 도구와 Benetti-Pinto 등[14]이 사용한 World Health Organization 삶의 질 도구에 따른 차이를 배제할 수 없으므로 추후 반복 연구가 필요하다. 조기폐경 여성은 폐경 후 여성성의 상실과 불임 사실에 힘들어하고[8] 이행기에 신체 기능의 저하로 삶의 질이 저하되지만[15], 본 연구의 대상자 대부분이 폐경된 지 10년 이상 지났으므로 시간이 경과되면서 폐경의 충격이 삶의 질에 미치는 영향이 둔화된 것으로 추정된다. 건강한 폐경기의 개념은 폐경기 이행부터 폐경 후기까지 자연폐경, 조기폐경 여성을 포함한 모든 여성에게 적용되는 것으로 신체적, 심리적, 사회적 기능에 대한 주관적인 지각 정도와 관련된다[27]. 그러므로 조기폐경 여성에게도 폐경으로 인한 변화에 잘 적응할 수 있도록 자기 관리 방법을 지원해야 하며, 삶의 질 향상을 위해 관련 요인을 고려한 다차원적인 전략이 필요하다.

본 연구에서 조기폐경군에서 삶의 질 관련 요인으로는 주관적 건강 상태와 우울 증상이 확인되었다. Benetti-Pinto 등[14]의 연구에서도 조기폐경 여성의 신체적 건강과 삶의 기쁨과 의미, 부정적인 느낌의 심리적 영역을 삶의 질 영향 요인으로 보고하여 본 결과를 뒷받침하였다. 선행연구에서 조기폐경군에서 자신의 건강 상태를 나쁘다고 인식하는 비율이 높았으며[16], 조기폐경 여성은 폐경 상황에 따른 우울과 대인 관계의 위축으로 일상생활 활동이 저하되고[9], 신체적 기능과 삶의 질이 낮았다고[15] 보고하여 본 연구의 결과를 지지하였다. 조기폐경과 건강 상태의 문제는 폐경으로 인한 에스트로겐 수준의 저하와 관련이 있을 수 있고[28], 대상자의 낮은 건강 상태에 대한 주관적인 인식은 객관적인 건강 상태, 즉 질병과 정신적 불건강으로 이어질 수 있다[24]. 따라서 조기폐경 여성의 현재 신체 상태에 대한 주관적 인식과 신체 상태, 우울 정도를 조기에 사정하고 필요한 경우 심리사회적인 중재를 제공하는 삶의 질 향상 프로그램 개발이 필요하다.

한편 본 연구에서 정상폐경군의 삶의 질 관련 요인으로는 유산소 신체활동 실천, 주관적 건강 상태, 스트레스, 우울 증상이 확인되었다. 선행연구에서 정상폐경 여성의 신체활동 유무[22], 우울과 스트레스 정도[29]가 삶의 질 저하에 영향을 미친다고 하여 본 연구의 결과를 뒷받침하였다. 신체적 활동은 자기 관리, 불안과 우울의 삶의 질과 관련이 있다고 보고되었으므로, 정상폐경 여성이 꾸준히 주 3회의 규칙적인 유산소 운동을 유지하도록 교육하여 신체적 건강 상태를 개선하고 스트레스, 우울을 해소할 수 있도록 삶의 질 향상 전략을 마련하는 것이 좋겠다.

본 연구는 조기폐경군과 정상폐경군의 삶의 질 관련 요인의 차이를 조사하였으며, 주관적 인식과 우울 증상은 두 군 모두에서 삶의 질 관련 요인으로 나타났으나 영향력은 조기폐경군에서 더 높았다. 이러한 결과는 정상폐경군보다는 조기폐경군의 우울 증상이 삶의 질에 더 크게 영향을 미칠 수 있음을 시사한다. 조기폐경 여성에게는 폐경 후 동반 질환 조절을 포함한 추후 건강 관리를 통해 주관적인 건강 상태 인식을 향상시키고 우울 증상에 적극적으로 개입하는 것이 삶의 질 향상에 도움이 될 것으로 생각한다. 정상폐경군에서는 이 두 요인 외에도 유산소 신체활동 실천 스트레스가 영향요인임을 확인하였다. 정상폐경군에서는 유산소 운동 실천 권장, 일상적인 스트레스 관리 등의 자기관리 방법에 대한 교육 등이 삶의 질을 향상시키는 데 도움이 될 것으로 생각된다. 더불어 삶의 질 향상 프로그램을 개발하여 적용할 때는 연령과 교육수준, 폐경 후 기간을 고려하여 내용을 구성하고 교육하는 것이 필요하며, 삶의 질 향상에 대한 추이를 관리하는 체계의 마련도 필요하다

본 연구는 국민건강영양조사 자료를 활용하였으므로 건강 형태 특성, 신체건강과 정신건강 특성에 따른 조기폐경군과 정상폐경군의 삶의 질 정도와 관련 요인을 파악하는 데 제한적이다. 또한 조기폐경 여부에 따른 삶의 질을 운동 능력, 자기 관리, 일상생활, 통증/불편, 불안/우울에 국한하여 분석하였으므로 확대 해석하기에는 제한적이다. 폐경 후 약 10년을 골다공증과 심혈관질환 위험도가 증가하는 시기로 보고하여[30], 본 연구에서는 70세 이상군이 1565명으로 폐경여성의 폐경 후 삶의 질 관련요인에 대해 확대 해석하기에는 제한적이다. 추후 조기폐경군과 정상폐경군의 삶의 질에 영향을 미치는 매개변수를 파악하고, 인공폐경에 따른 조기폐경 여성의 정서 상태와 삶의 질을 파악하는 연구가 필요하다.

이상의 연구 결과에서 우리나라 여성의 조기폐경 여부에 따른 삶의 질은 차이가 없었으나 삶의 질 관련 요인들은 차이가 있었다. 즉, 주관적 건강 상태와 우울 증상은 조기폐경 여성과 정상폐경 여성 모두에서 삶의 질 관련 요인으로 확인되었고, 정상폐경 여성에서는 유산소 신체활동 실천 여부와 스트레스도 삶의 질과 관련이 있음을 확인하였다. 정상폐경 여성뿐 아니라 조기폐경 여성에게도 폐경으로 인한 변화에 적응할 수 있도록 자기 관리 방법을 지원해야 하며, 폐경 여성의 삶의 질 향상을 위해 주관적인 건강 상태와 우울 증상을 고려한 다차원적 전략이 필요하다.

본 연구 결과를 토대로 조기폐경 및 정상폐경 여성의 폐경 후 기간에 따른 삶의 질 변화 양상과 영향요인을 비교하는 연구를 제언한다. 그리고, 조기폐경 여부를 고려하여 폐경 후 건강 관리 방법에 대한 정보를 제공하고, 우울과 같은 부정적인 정서 상태에 대해 상담을 받을 수 있도록 지원해야 한다.

## Figures and Tables

**Table 1.** General characteristics of premature menopausal women and normal menopausal women in the KNHANES 2014–2017 (N=5,910)

Characteristics	Categories	n (%)^†^			F^‡^(*p*)
		Total (n=5,910)	Premature menopause (n=322)	Normal menopause (n=5,588)	
Age (year)	<50	222 (5.3)	51 (20.7)	171 (4.4)	45.15 (<.001)
	50–59	2,017 (41.0)	87 (33.3)	1,930 (41.5)	
	60–69	2,106 (31.1)	74 (18.0)	2,032 (31.9)	
	≥70	1,565 (22.6)	110 (28.0)	1,455 (22.2)	
Marriage	Yes	5,856 (99.1)	317 (98.2)	5,539 (99.2)	2.68 (.102)
	No	54 (0.9)	5 (1.8)	49 (0.8)	
Occupation	Office worker	382 (7.0)	26 (10.2)	356 (6.8)	1.70 (.182)
	Manual occupation	2,249 (38.4)	124 (37.5)	2,125 (38.5)	
	Unemployed	3,279 (54.6)	172 (52.3)	3,107 (54.7)	
Income (n=5,888)^§^	Low	1,749 (26.7)	106 (29.2)	1,643 (26.6)	0.53 (.660)
	Middle-low	1,610 (26.2)	85 (23.3)	1,525 (26.3)	
	Middle-high	1,275 (23.2)	64 (24.2)	1,211 (23.2)	
	High	1,254 (23.9)	67 (23.3)	1,187 (23.9)	
Education (n=5,902)^§^	Elementary school	2,772 (42.1)	178 (46.1)	2,594 (41.9)	10.12 (.018)
	Middle school	1,028 (18.0)	29 (10.3)	999 (18.5)	
	High school	1,411 (27.1)	78 (28.4)	1,333 (27.0)	
	University or higher	691 (12.8)	37 (15.2)	654 (12.6)	
Region	City	2,681 (47.6)	144 (46.0)	2,537 (47.7)	0.25 (.616)
	Country	3,229 (52.4)	178 (54.0)	3,051 (52.3)	
Cause of menopause	Natural menopause	5,064 (84.9)	118 (34.7)	4,946 (87.9)	514.39 (<.001)
	Artificial menopause	846 (15.1)	204 (65.3)	642 (12.1)	
Postmenopausal period (year)	<10	2,211 (44.2)	24 (8.6)	2,187 (46.3)	141.73 (<.001)
	≥10	3,699 (55.8)	298 (91.4)	3,401 (53.7)	

† Weighting adjustment.

‡ Calculated by the Rao-Scott χ^2^ test.

§ Excluding non-response.

**Table 2.** Health-related behaviors, physical health, and psychological health in premature menopausal women and normal menopausal women (N=5,910)

Variable	Categories	n (%)^†^			F^‡^(*p*)
		Total	Premature menopause	Normal menopause	
*Health-related behaviors*					
Smoking	No	5,652 (96.0)	294 (89.8)	5,358 (96.4)	21.72 (<.001)
	Yes	193 (4.0)	24 (10.2)	169 (3.6)	
Heavy episodic drinking	No	5,362 (89.4)	290 (89.8)	5,072 (89.4)	0.05 (.823)
	Yes	548 (10.6)	32 (10.2)	516 (10.6)	
Aerobic physical activity prevalence	Yes	2,282 (41.2)	105 (26.2)	2,177 (41.5)	2.59 (.107)
	No	3,589 (58.8)	213 (63.8)	3,376 (58.5)	
*Physical health*					
Subjective health	Very good or good	1,144 (19.6)	49 (16.6)	1,095 (19.7)	4.50 (.011)
	Moderate	3,055 (52.8)	148 (47.1)	2,907 (53.1)	
	Very bad or bad	1,709 (27.6)	125 (36.3)	1,584 (27.2)	
Hypertension	No	3,152 (57.0)	165 (58.0)	2,987 (56.9)	0.11 (.742)
	Yes	2,754 (43.0)	157 (42.0)	2,597 (43.1)	
Diabetes mellitus	No	5,072 (86.8)	258 (82.2)	4,814 (87.0)	5.04 (.025)
	Yes	837 (13.2)	64 (17.8)	773 (13.0)	
*Psychological health*					
Stress	No	4,425 (75.0)	235 (72.8)	4,190 (75.1)	0.53 (.461)
	Yes	1,421 (25.0)	83 (27.2)	1,338 (24.9)	
Depression symptom	No	5,378 (91.4)	282 (87.2)	5,096 (91.7)	6.56 (.010)
	Yes	531 (8.6)	40 (12.8)	491 (8.3)	

† Analyzed excluding non-response: weighting adjustment.

‡ Calculated by the Rao-Scott χ^2^ test.

**Table 3.** Quality of life in premature menopausal women and normal menopausal women (N=5,910)

Variable	Mean±SD			t	*p*
	Total	Premature menopause	Normal menopause		
Mobility	1.26±0.01	1.35±0.07	1.26±0.01	1.43	.153
Self-care	1.07±0.01	1.13±0.06	1.06±0.01	1.10	.272
Usual activities	1.15±0.01	1.22±0.06	1.14±0.01	1.33	.185
Pain/discomfort	1.39±0.01	1.45±0.07	1.39±0.01	0.36	.361
Anxiety/depression	1.17±0.01	1.25±0.07	1.17±0.01	1.24	.216
Quality of life	0.91±0.01	0.89±0.01	0.91±0.01	–1.57	.118

**Table 4.** Factors related to quality of life according to the characteristics of premature menopausal women and normal menopausal women (N=5,910)

Variable	Categories	Premature menopause		Normal menopause	
		OR (95%CI)	*p*	OR (95%CI)	*p*
*Health-related behaviors*					
Smoking	No	1		1	
	Yes	0.56 (0.20–1.61)	.167	1.40 (0.86–2.25)	.177
Heavy episodic drinking	No	1		1	
	Yes	2.99 (0.84–10.71)	.430	0.95 (0.71–1.29)	.759
Aerobic physical activity prevalence	Yes	1		1	
	No	1.19 (0.53–2.68)	.734	1.35 (1.12–1,63)	.001
*Physical health*					
Subjective health	Very good or good	1		1	
	Moderate	1.71 (0.43–6.75)	.496	2.28 (1.70–3.05)	<.001
	Very bad or bad	5.49 (1.49–20.28)	.013	9.42 (6.96–12.74)	<.001
Hypertension	No	1		1	
	Yes	1.53 (0.62–3.75)	.401	1.05 (0.87–1.27)	.599
Diabetes mellitus	No	1		1	
	Yes	0.69 (0.25–1.88)	.502	0.86 (0.68–1.10)	.226
*Psychological health*					
Stress	No	1		1	
	Yes	2.21 (0.97–5.03)	.068	1.62 (1.35–1.94)	<.001
Depression symptom	No	1		1	
	Yes	4.25 (1.72–10.48)	.002	1.67 (1.27–2.20)	<.001
Likelihood ratio *x*^2^		114.07		1268.90	
*p*		<.001		<.001	
Nagelkerke R^2^		.31		22	
Max-rescaled R^2^		.47		.34	

CI: confidence interval; OR: odds ratio.
